# Oncogene-Induced Replication Stress Drives Genome Instability and Tumorigenesis

**DOI:** 10.3390/ijms18071339

**Published:** 2017-06-22

**Authors:** Dan Sarni, Batsheva Kerem

**Affiliations:** Department of Genetics, The Life Sciences Institute, The Hebrew University, 91904 Jerusalem, Israel; dan.sarni@mail.huji.ac.il

**Keywords:** oncogene, DNA replication, replication stress, genomic instability, cancer

## Abstract

Genomic instability plays a key role in driving cancer development. It is already found in precancerous lesions and allows the acquisition of additional cancerous features. A major source of genomic instability in early stages of tumorigenesis is DNA replication stress. Normally, origin licensing and activation, as well as replication fork progression, are tightly regulated to allow faithful duplication of the genome. Aberrant origin usage and/or perturbed replication fork progression leads to DNA damage and genomic instability. Oncogene activation is an endogenous source of replication stress, disrupting replication regulation and inducing DNA damage. Oncogene-induced replication stress and its role in cancer development have been studied comprehensively, however its molecular basis is still unclear. Here, we review the current understanding of replication regulation, its potential disruption and how oncogenes perturb the replication and induce DNA damage leading to genomic instability in cancer.

## 1. Introduction

Genomic instability, found in most cancers, is considered one of the hallmark characteristics of cancer cells. Furthermore, it is regarded as a major driver of tumorigenicity, evident already in precancerous lesions [1,2,3,4,5]. Several factors have been suggested to underlie genomic instability found in cancer cells such as telomere attrition [6], oxidative stress [7] and defective DNA damage repair [8]. However, these factors mainly contribute to genomic instability apparent in advanced stages of cancer development. In early stages of tumorigenicity, genomic instability was found preferentially in fragile sites, which are genomic regions sensitive to replication perturbation [9], implying that perturbed replication may underlie the early genomic instability found in cancers [4,5]. Replication stress, which is characterized by deceleration of replication fork progression and an increase in stalled and collapsed forks [10], was found to induce genomic instability and promote tumorigenesis. Aphidicolin, a DNA polymerase inhibitor, causing replication stress when used in low concentrations, was found to induce micro-deletions resembling those found in tumors [11]. In addition, hydroxyurea treatment inhibits the ribonucleotide reductase (RNR), leading to low deoxynucleotide triphosphate (dNTP) levels promoted tumor formation in mice [12], thus strengthening the causative link between replication stress and tumorigenicity.

Oncogene activation was found to induce endogenous replication stress leading to genomic instability and DNA damage response (DDR) in early cancer stages [13,14]. This DDR activation could lead to proliferation arrest either in the form of senescence or apoptosis, forming a tumorigenic barrier [13,14]. However, such barriers could be bypassed by a defective DDR, usually caused by impairment of the p53 tumor suppressor pathway. Thus, replication-induced genomic instability could lead to such alterations of cellular processes promoting tumorigenicity [15].

Despite the accumulated evidence indicating that oncogene-induced replication stress plays a crucial role in early tumorigenesis, its molecular basis is still largely unclear. Several mechanisms were suggested to underlie oncogene-induced replication stress including: cell cycle regulation enforcing proliferation, deregulating origin licensing and activation as well as endangering faithful replication fork progression and genome duplication. Recently, it has been reported that different oncogenes induce unique repertoires of recurrent chromosomal breaks expressed as fragile sites [16]. This implies that oncogene-induced instability is oncogene-dependent, possibly due to the varying cellular effects each oncogene enforces upon a cell. In this review, we will summarize the current understanding of DNA replication, regulation of origins, and fork progression. We will discuss suggested mechanisms underlying oncogene-induced replication stress and how such replication stress may induce genomic instability in cancer.

### 1.1. DNA Replication Regulation—From Licensing to Termination

DNA replication is highly regulated to ensure the faithful duplication of the genome [17]. Cells start preparing for DNA replication in G1-phase of the cell cycle when potential origins are licensed [17]. Origin licensing is the process of assembling the pre-replication complex (pre-RC) at replication origins by loading the origin recognition complex (ORF) onto the chromatin, followed by Cdc6- and Cdt1 binding, which consequently recruit the mini-chromosome maintenance (MCM) complex [17,18,19,20]. The MCM complex, which serves as the main replicative helicase, is loaded as an inactive double hexamer at first. Many more origins are licensed than activated, as the number of active origins in S-phase is lower than the number of licensed replication origins. This provides a safety network of potential origins for activation when needed, such as in the case of replication stress [21,22,23]. Which origins will be activated, and how are they selected for activation, is still largely unclear. However, the process of origin activation is better understood. At the G1/S transition cyclin-dependent kinase (CDK) and DBF4-dependent kinase (DDK) activate the MCM complex which subsequently recruits Cdc45 and the GINS (Sld5-Psf1-Psf2-Psf3) complex, forming the CMG (Cdc45-MCM-GINS) complex [24,25,26]. After CMG assembly and activation, it unwinds the double-stranded DNA to initiate DNA replication. Replication terminates when replisomes from adjacent origins converge. Recently, it has been shown that CMG unloading occurs after the final ligation step of the leading and lagging strands from opposing forks [27]. Furthermore, CMG dissociation is not required for completion of DNA synthesis, thus suggesting that the CMGs pass each other and dissociate from double-stranded DNA, which may prevent unwanted CMG unloading from active replication forks [27].

To ensure that the genome is duplicated only once every cell cycle, origin licensing and initiation are temporally separated due to CDK activity oscillations [28]. Origin licensing is restricted to G1-phase, and at the onset of S-phase CDK activity increases, which results in origin activation. CDKs also inhibit assembly of the pre-RCs, preventing re-licensing of activated origins. CDK phosphorylates Cdt1 and Orc1, targeting them for degradation [29,30,31]. Furthermore, Cdt1 loading onto chromatin is inhibited by geminin, which is present from S through M phase [32]. Thus, origin activation is tightly regulated to prevent inappropriate and potentially harmful re-usage of replication origins, re-replication.

Replication is temporally programmed so that origins fire in a scheduled manner throughout S-phase [33]. The nature of timely activation of origins is largely unknown; however, chromatin modifications, transcriptional activation and, most strikingly, nuclear positioning may all play a role. Thus, disruption of any of these factors associated with the replication timing program or perturbation of any stage in DNA replication may lead to genomic instability.

### 1.2. Mechanisms Underlying Oncogene-Induced Replication Stress

#### 1.2.1. Paucity of Origin Activation

Oncogene activation may disrupt cell cycle regulation thereby forcing cell proliferation. Such unscheduled progression through the cell cycle may alter the replication program and result in replication stress. As mentioned above, origin licensing and activation are inversely controlled by CDK activity [28]. Under normal conditions, not all licensed origins are activated, and the rest, termed “dormant origins”, serve as potential backups for replication initiation under stress conditions such as replication stress [34]. Moderate reduction in pre-RCs, for instance, can lead to decreased origins being activated during DNA replication, possibly by depleting the backup pool of dormant origins when needed. Indeed, depletion of MCMs to levels that did not affect replication dynamics rendered cells sensitive to replication stress and led to fork stalling and DNA damage [35,36]. Interestingly, overexpression of the oncogene cyclin E, which promotes G1/S transition by binding to CDK2, impaired MCM2–7 binding to the chromatin resulting in a reduced number of activated origins in early S-phase [37]. Ectopic expression of other oncogenes such as *HPVE7*, *KRAS* and *Myc* was reported to sensitize cells to depletion of pre-RC components such as MCMs, Cdc6 and Orc1, respectively [38,39,40]. Shortage in replication origins may also result from inhibition of origin activation. Indeed, the oncogene Mdm2 was shown to inhibit origin activation in early S-phase by inducing S-phase checkpoint response in the absence of p53 [41]. Altogether, oncogene activation induces perturbed replication dynamics characterized by paucity of origin activation either due to reduction in licensed origins or through direct inhibition of origin firing. This perturbed replication impairs the faithful duplication of the genome, leading to genomic instability.

#### 1.2.2. Origin Hyper-Activation

Disrupting the cell cycle regulation may lead to unscheduled origin activation. Alteration of origin usage can result in origins firing earlier than programmed, origin re-activation within the same cell cycle, and a general increase in origin activation. Hyper-origin activation can induce DNA damage and promote genomic instability during tumor development, as demonstrated by the overexpression of licensing factors [42]. Overexpression of the human origin licensing factors Cdt1 or Cdc6 in mice led to increased origin activation and induced the DDR [42]. In addition, these factors were found abnormally over-accumulated in epithelial lesions from the stage of dysplasia, suggesting they may play a role in tumor development [42]. Oncogenes may promote proliferation by driving cell cycle progression and by deregulating gene expression. The *Myc* oncogene is a master regulator of cell proliferation, which regulates the expression of many proliferative genes, including dNTP biosynthesis factors, replication factors, CDKs etc. [43]. Myc was also found to interact directly with pre-RC components and co-localize with replication foci in early S phase [44]. Thus, *Myc* overexpression found in many cancers may promote origin activation both transcriptionally and directly by binding in the vicinity of origins.

Local origin hyper-activation can be detected by analysis of single replicating DNA fibers using DNA combing, where it is manifested as reduced inter-origin distances. Such a decrease in inter-origin distances was reported in cells overexpressing the oncogenes *cyclin E*, *HPV E6* and *E7*, *RAS* and *Myc* [45,46,47]. These oncogenes also promote proliferation by forcing G1/S transition, which may indicate deregulation of CDK activity in these cases. Alternatively, hyper-origin activation may result from reduced replication fork rate [48]. Dormant origins are activated following inhibition of replication fork progression [35,49]; thus, local increase in origin usage may result from replication fork slowing and stalling.

Re-replication may occur if origins are re-licensed during S phase, consequently leading to origin activation more than once per cell cycle. Thus, re-replication endangers the proper duplication of the genome. Therefore, licensing is highly regulated to prevent such deleterious events, as mentioned above. *Cdt1* overexpression induced re-replication in cancer, but not in normal cells [50]. It was also reported that RAS-induced senescent cells display re-replication [14]. Recently, p53 independent-p21 overexpression has been reported to possess an oncogenic effect by inducing deregulation of licensing factors triggering replication stress [51]. Interestingly, following p21 ectopic expression, cells underwent cell cycle arrest, but a subpopulation of p21-expressing cells escaped with upregulated licensing factors, which led to re-replication, increased DNA damage and genomic instability, and increased chemoresistance [51]. Altogether, origin hyper-activation is a common characteristic of oncogene-induced replication stress, and may underlie oncogene-induced genomic instability.

#### 1.2.3. Perturbation of Replication Fork Elongation

In addition to origin deregulation, oncogene activation also affects replication fork progression. One of the hallmark characteristics of replication stress is replication fork deceleration, which may lead to fork stalling and, if not recovered, to fork collapse [10]. Arrested replication forks are deleterious to genome integrity and cell faith. Several mechanisms have been reported to aid in recovering replication and restraining potential damage, such as replication restart by homologous recombination and template switching, translesion polymerization, and replication fork remodeling [52]. However, not all arrested forks are efficiently repaired, thus leading to DNA damage and genomic instability.

Replication fork deceleration has been reported following oncogenes overexpression, such as *RAS*, *cyclin E* and *HPV E6/E7* [45,46,47,53]. This replication rate decrease is accompanied by decreased inter-origin distances, implying activation of dormant origins and increased fork asymmetry, indicating fork stalling. Replication fork deceleration and stalling compromise the faithful duplication of the genome, as it may lead to incomplete replication. Such under-replicated regions may lead to genomic instability. One example is the expression of fragile sites in metaphase chromosomes. Fragile sites are genomic regions sensitive to replication stress, caused by decelerated replication fork progression and inefficient origin activation [54,55]. The attempt of cells to activate additional origins in order to compensate for the slow replication may not be sufficient, and cells enter mitosis with under-replicated DNA.

*Cyclin E* and *HPV E6/E7* overexpression were found to promote proliferation without activating nucleotide biogenesis genes, leading to insufficient levels of dNTPs and resulting in replication stress [45]. Exogenous supplementation of nucleosides rescued the slow replication fork rate and the DNA damage. Interestingly, *Myc* overexpression in these cases upregulated the expression of nucleotide biogenesis genes, and reduced replication stress as well [45]. Recently, *RAS*-induced replication fork deceleration was also partially rescued by exogenous nucleoside supplementation [47], indicating that shortage of dNTPs maybe at the basis of oncogene-induced replication stress. Furthermore, environmental alterations may enhance the oncogene-induced replication stress. Recently, it was reported that deficiency of folate, a micronutrient essential for dNTP biosynthesis, enhanced *RAS*- and *cyclin E*-induced replication stress and increased tumorigenicity in mice [56]. Thus, suggesting that the extent of replication stress plays a major role in cancer development induced by both genetic and non-genetic factors.

*Cyclin E*-induced replication stress has also been reported to be rescued by inhibition of the origin initiation factors Cdc6 and Cdc7/CDK [46]. Thus, *cyclin E*-induced replication stress may result from insufficient levels of dNTPs at replication onset or from origin hyper-activation. These results may not be mutually exclusive, as *cyclin E* may drive both unscheduled G1/S transition with insufficient dNTPs and hyper-activate origins in parallel pathways. It is worth noting that replication stress may lead to shortage of additional replication factors during DNA replication, such as replication protein A (RPA) [57].

In addition to replication factor exhaustion, replication may collide with obstacles along the DNA. Transcription, occurring during S-phase, was reported to be spatially segregated from replication domains [58]. Conversely, early replication was found to correlate with transcription [33]. It has been reported that abnormal accumulation of DNA-RNA hybrids, termed “R-loops”, leads to replication perturbation and DNA damage [59,60]. Interestingly, replication stress induced by both *cyclin E* and *RAS* was also rescued by transcription inhibition [46,47], indicating that oncogene-induced replication perturbation may result from oncogene-induced transcriptional deregulation. Since these oncogenes promote G1/S transition and origin activation as well as transcriptional control, it is possible that uncoordinated replication and transcription will induce aberrant collisions between these machineries. Altogether, the mechanisms suggested to underlie oncogene-induced replication stress may vary among oncogenes and cellular environments as different oncogenes affect the expression of different genes and cellular pathways.

### 1.3. Replication-Induced Genomic Instability in Cancer

Chromosomal instability, leading to changes in chromosome structure and number, is prevalent in most human cancers, and is a cause of intra-tumor heterogeneity and aneuploidy [61,62]. Replication stress has been reported to induce chromosomal aberrations such as acentric chromosomal fragments and anaphase bridges in colorectal cancer cells, possibly promoting cancer development [63]. Chromosomal instability induced by impaired replication may result from incomplete replication, either due to fork stalling or to paucity of origin activation. Interestingly, even a significant reduction in replication rate could be tolerable and enable mitotic onset. However, cell cycle progression with under-replicated DNA may lead to anaphase bridges, extra centromeres, chromosomal breaks and even metaphase arrest [64,65]. Furthermore, replication stress has been reported to induce the formation of micronuclei, aberrant nuclear structures physically separated from the main nucleus [66]. Interestingly, it has been suggested that under-replication of the DNA within micronuclei leads to extensive chromosomal rearrangements which may lead to the formation of numerous chromosomal rearrangements in a single catastrophic event defined as chromothripsis [67,68]. Thus, replication stress may drive the massive chromosomal rearrangements apparent in cancers.

Common fragile sites (CFSs), which are induced by replication stress, were found to correlate with breakpoints in tumors, deletions of tumor suppressor genes and amplification of oncogenes, promoting tumorigenesis [69,70,71,72]. Furthermore, genomic alterations are more common in CFSs as result of oncogene-induced replication stress in pre-neoplastic lesions [73]. Importantly, processing of CFSs in mitosis by MUS81–EME1 and ERCC1, specific DNA structure nucleases, has been reported to enable the resolution of sister chromatids and allowed correct chromosomal segregation [74,75]. CFSs are a manifestation of under-replicated DNA in mitosis. Recently, DNA synthesis has been reported at CFSs during mitosis, which is dependent on POLD3 and MUS81–EME1 activity [76]. Interestingly, inhibition of DNA synthesis during mitosis led to chromosome missegregation and non-disjunction, increasing ultrafine anaphase bridges, but reducing CFS expression [76]. Altogether, these results indicate that if aberrant replication is not corrected, even as late as mitosis, it may serve as the basis for chromosomal instability.

## 2. Conclusions

**Figure 1:**
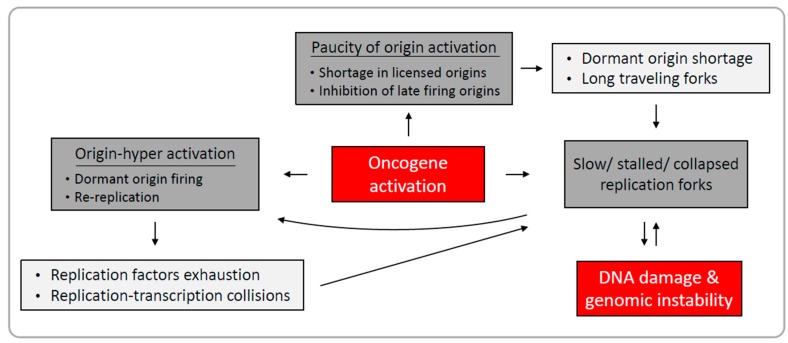
Oncogene-induced replication stress leads to DNA damage and genomic instability. Oncogene activation disrupts replication regulation leading to slow and stalled replication forks, hyper-activation and/or paucity of origin activation. Such deregulated replication causes DNA damage. Dark grey boxes are manifestations of deregulated replication, light grey boxes are the potential outcomes of origin deregulation.

Oncogene-induced replication stress plays a major role in early cancer development. Several forms of replication perturbation leading to genomic instability have been reported following oncogene activation. These include slowed and arrested replication fork progression, as well as deregulated origin licensing and activation. Oncogenes may affect different cellular processes promoting various forms of replication stress (Figure 1). In addition, a single oncogene may drive replication perturbation by several mechanisms, indicating that the nature of oncogene-induced replication stress is complex. Although replication stress induced by oncogene activation has been comprehensively studied, there is much more to explore. A better understanding of oncogene-induced replication stress is crucial for our understanding of replication-induced genomic instability and tumorigenesis. It is important to note that, while replication-induced genomic instability has been closely studied in the early stages of cancer development, the precise profile of replication dynamics in later stages of tumor development may be altered as cancerous cells adapt to the stressful environment, and additional stress factors may come into play. Such alterations may have important implications when replication stress is considered for therapy.
